# BMP and TGFbeta pathways in human central chondrosarcoma: enhanced endoglin and Smad 1 signaling in high grade tumors

**DOI:** 10.1186/1471-2407-12-488

**Published:** 2012-10-22

**Authors:** Stephane Boeuf, Judith VMG Bovée, Burkhard Lehner, Brendy van den Akker, Maayke van Ruler, Anne-Marie Cleton-Jansen, Wiltrud Richter

**Affiliations:** 1 Research Centre for Experimental Orthopaedics, Department of Orthopaedics, Trauma Surgery and Paraplegiology, Heidelberg University Hospital, Schlierbacher Landstrasse 200a, 69118, Heidelberg, Germany; 2 Department of Pathology, Leiden University Medical Center, Albinusdreef 2, 2333 ZA, Leiden, The Netherlands; 3 Division of Orthopaedic Oncology, Department of Orthopaedics, Trauma Surgery and Paraplegiology, Heidelberg University Hospital, Schlierbacher Landstrasse 200a, 69118, Heidelberg, Germany

**Keywords:** Conventional central chondrosarcoma, Bone tumor, Chondrogenic differentiation, Bone morphogenic proteins, Transforming growth factor β

## Abstract

**Background:**

As major regulators of normal chondrogenesis, the bone morphogenic protein (BMP) and transforming growth factor β (TGFB) signaling pathways may be involved in the development and progression of central chondrosarcoma. In order to uncover their possible implication, the aim of this study was to perform a systematic quantitative study of the expression of BMPs, TGFBs and their receptors and to assess activity of the corresponding pathways in central chondrosarcoma.

**Methods:**

Gene expression analysis was performed by quantitative RT-PCR in 26 central chondrosarcoma and 6 healthy articular cartilage samples. Expression of endoglin and nuclear localization of phosphorylated Smad1/5/8 and Smad2 was assessed by immunohistochemical analysis.

**Results:**

The expression of TGFB3 and of the activin receptor-like kinase ALK2 was found to be significantly higher in grade III compared to grade I chondrosarcoma. Nuclear phosphorylated Smad1/5/8 and Smad2 were found in all tumors analyzed and the activity of both signaling pathways was confirmed by functional reporter assays in 2 chondrosarcoma cell lines. Immunohistochemical analysis furthermore revealed that phosphorylated Smad1/5/8 and endoglin expression were significantly higher in high-grade compared to low-grade chondrosarcoma and correlated to each other.

**Conclusions:**

The BMP and TGFβ signaling pathways were found to be active in central chondrosarcoma cells. The correlation of Smad1/5/8 activity to endoglin expression suggests that, as described in other cell types, endoglin could enhance Smad1/5/8 signaling in high-grade chondrosarcoma cells. Endoglin expression coupled to Smad1/5/8 activation could thus represent a functionally important signaling axis for the progression of chondrosarcoma and a regulator of the undifferentiated phenotype of high-grade tumor cells.

## Background

Conventional central chondrosarcomas are cartilaginous tumors which arise centrally within the medullar cavity of bone. They represent 75% of all malignant cartilage tumors. Low-grade chondrosarcoma displays a hyaline cartilage matrix with low cell density, and an abundance of hyaline cartilage matrix, no mitoses and cells with a chondrocyte-like morphology. While these tumors generally do not metastasize, they can progress to high-grade chondrosarcomas which are characterized by a muco-myxoid matrix, a high density of cells with increased mitotic rates and elevated vascularization. At the periphery of the lobules of high-grade chondrosarcoma, cells may become spindle-shaped [1]. These tumors often metastasize, are considered resistant to chemotherapy and radiotherapy and the 10 years survival rate is only 29% for grade III chondrosarcoma [2].

The morphology of the cells and the composition of the matrix in central chondrosarcoma suggest parallels between differentiation stages of tumor cells and of normal chondrocytes [3]. Gene expression profiles have indicated that during progression chondrosarcoma cells shift from a differentiated state in low-grade tumors to a state more similar to early chondrogenic differentiation stages of mesenchymal precursor cells in high-grade tumors [4]. The correlation of the differentiation stage of chondrosarcoma cells to the degree of malignancy of the tumors indicates that signaling pathways that control normal chondrogenesis may have a regulatory function in the progression of these tumors.

Bone morphogenic protein (BMP) and transforming growth factor β (TGFβ) signaling is one of the crucial pathways controlling chondrogenic differentiation in the normal growth plate [5]. The main paracrine factors of the TGFβ superfamily relevant for cartilage and bone formation are BMP2, BMP4, BMP6, BMP7, TGFβ1, TGFβ2 and TGFβ3. Signaling is initiated when BMPs bind to the type II receptor BMPRII and TGFβ molecules to TGFBRII. These receptors are transmembrane serine/threonine kinases which upon binding of a ligand recruit the type I receptors ALK1, ALK2, ALK3 or ALK6 for BMPRII and ALK1 or ALK5 for TGFBRII, leading to phosphorylation and activation of the type I receptor kinases. The activated type I receptors in turn phosphorylate intracellular Smad molecules which translocate in the nucleus and modulate the expression of target genes. The activation of ALK1/2/3/6 induces the phosphorylation of Smad1, Smad5 and Smad8, while ALK5 induces Smad2 and Smad3 [6,7]. BMPs thus activate Smad1/5/8 while TGFβ, depending on the type I receptor recruited, can activate either Smad2/3 or Smad1/5/8. In endothelial cells and chondrocytes, the TGFβ/ALK1/Smad1 signaling axis appears to be favored in presence of the TGFβ co-receptor endoglin, also known as CD105 [7,8].

As shown by detection of nuclear Smad proteins, the TGFβ and BMP signaling pathways are active in most cells of the growth plate and they are controlled by tight temporal and local patterns of expression of the factors of the TGFβ superfamily and of their receptors [9]. In central chondrosarcoma TGFβ signaling is active according to detection of nuclear phosphorylated Smad2. A role of this pathway in tumor progression was suggested as PAI1, a target gene of TGFβ/Smad2/3, showed higher levels in high grade tumors [10]. In an immunohistochemical study, a correlation of TGFβ1 and TGFβ2 to the grade of chondrosarcoma has been described [11]. In contrast to these results suggesting that TGFβ signaling could be involved in chondrosarcoma progression, data demonstrating active BMP signaling in chondrosarcoma tissue are lacking. While one immunohistochemical study found no BMPs in human conventional chondrosarcoma tissue [12], one RT-PCR based gene expression analysis detected expression of BMP2, 4, 6 and BMPRII [13]. The migratory effect of BMP2 on chondrosarcoma cell lines, however, suggests a role of BMP signaling in progression [14].

As major regulators of normal chondrogenesis, the BMP and TGFβ signaling pathways could play an active role in the progression of chondrosarcoma. Perturbations of these pathways are known to result in disorders ranging from vascular and skeletal disease to cancer [6]. In order to uncover a potential implication in chondrosarcoma, the aim of this project was to perform a systematic quantitative study of the expression of BMPs, TGFβs and their receptors and to assess activity of the corresponding signaling pathways in central chondrosarcoma cells.

## Results

### Expression of BMP and TGFÎ² ligands and receptors in central chondrosarcoma

**Figure 1:**
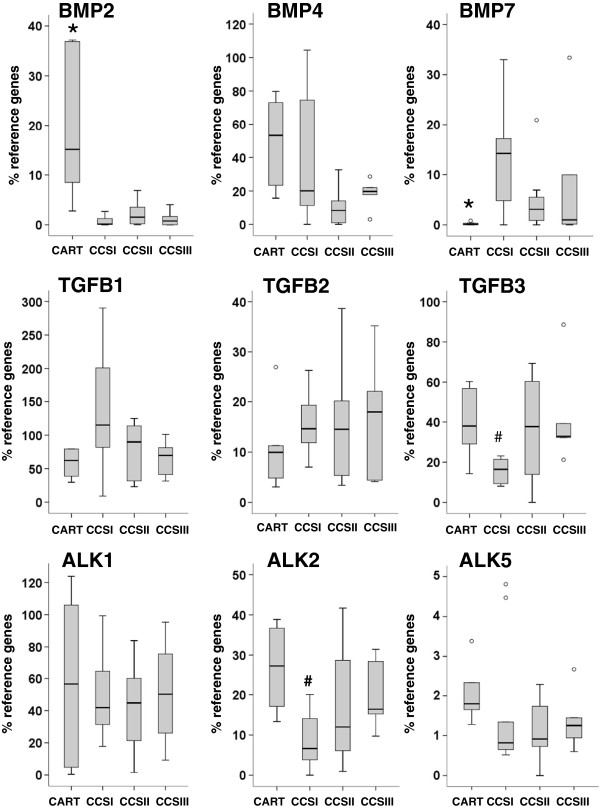
**Quantitative RT**-**PCR analysis of members of the BMP and TGFβ family in central chondrosarcoma.** Expression levels of BMP2, BMP4, BMP7, TGFB1, TGFB2, TGFB3, ACVRL1/ALK1, ACVR1/ALK2 and TGFBR1/ALK5 were assessed in normal cartilage (CART; n=6), grade I (CCSI; n=10), grade II (CCSII; n=10) and grade III (CCSIII; n=7) central chondrosarcoma samples and are shown as percentage of the mean expression levels of the reference genes. The median relative expression levels in chondrosarcoma and cartilage samples are represented by solid black lines, the boxes represent the interquartile range (IQR) extending between the 25th and 75th percentile and the whiskers extend to a maximum of 1.5 IQR. Outlier values are shown as empty circles. Statistical analysis is based on the non-parametric Mann–Whitney test after bonferroni correction (p<0.0125). # indicates significant difference in comparison to grade III; * indicates significant difference in comparison to all CCS.

The expression of genes for BMP and TGFβ ligands and receptors was measured in central chondrosarcoma and normal cartilage samples by quantitative RT-PCR (Figure 1). All of the genes analyzed were found to be expressed in chondrosarcoma samples. While among the ligands analyzed the BMP2, BMP4, BMP6, BMP7, TGFB1 and TGFB2 genes did not show significant differences between chondrosarcomas of different histological grades, TGFB3 was significantly higher expressed in grade III compared to grade I chondrosarcoma (2-fold, p=0.006). From the receptors analyzed, only the type I receptor ALK2 showed differential expression and was significantly higher in grade III than in grade I chondrosarcoma (2.5-fold, p=0.012).

Compared to normal cartilage, chondrosarcoma showed altered expression levels for BMP2 and BMP7. BMP2 was significantly higher expressed in normal cartilage samples than in chondrosarcoma (37.8-fold, p<0.001), while BMP7 was not detected or found at very low expression levels in normal cartilage samples and was significantly higher expressed in chondrosarcoma (29.4-fold, p=0.005). The expression of BMP6 (data not shown) was similar in all sample groups.

### Activity of Smad1/5/8 and Smad2 in central chondrosarcoma samples

**Figure 2:**
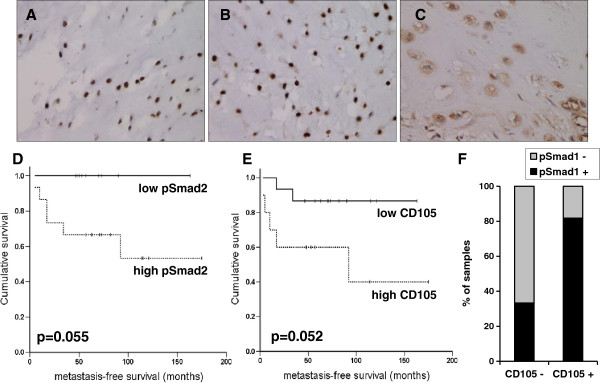
**Immunohistochemical analysis of phosphorylated Smad1**/**5**/**8**, **phosphorylated Smad2 and endoglin in central chondrosarcoma samples.** A grade III central chondrosarcoma with highly nuclear phosphorylated Smad1/5/8 (**A**) and highly nuclear phosphorylated Smad2 (**B**) is shown. **C**: grade II central chondrosarcoma with high endoglin expression. Note the positivity of the vessels in the right lower corner. Kaplan-Meier analysis of metastasis-free survival in relation to sum score of tumor biopsies for phosphorylated Smad2 (**D**) and endoglin / CD105 (**E**) is shown. High pSmad2 or CD105 designate tumor samples with a sum score higher than 3 for the corresponding antibody and survival is shown with a broken line. Survival for patients with low pSmad2 or CD105 is shown with a solid line. The p-values of corresponding log-rank tests are shown. **F**. Correlation between endoglin / CD105 expression and phosphorylated Smad1/5/8. The percentages of samples with highly (+) or low (−) phosphorylated Smad1/5/8 among samples with high (+) or low (−) endoglin / CD105 are shown.

In order to establish whether the BMP and TGFβ signaling pathways are active in central chondrosarcoma, the presence of nuclear phosphorylated Smad1/5/8 and Smad2 was evaluated by immunohistochemical analysis. Phosphorylated Smad1/5/8 and Smad2 was detected in all chondrosarcoma samples analyzed (Figure 2A, B). Highly phosphorylated Smad1/5/8, corresponding to a sum score higher than 3, was significantly more frequent in high-grade tumors compared to low grade while for highly phosphorylated Smad2 there was only a trend which did not reach significance (Table 1). There was a trend close to significance for a longer metastasis-free survival in patients with low phosphorylated Smad2, corresponding to a sum score lower or equal to 3 (p=0.055) (Figure 2D). This correlation was not independent from the histopathological grade of the tumors.

**Table 1:** Scoring results of the immunohistochemical staining in central chondrosarcoma

**Diagnosis**	**pSmad1**/**5**/**8**			**pSmad2**			**Endoglin** / **CD105**		
	**High**_**a**_	**%**	**p**-**value**_**b**_	**High**_**a**_	**%**	**p**-**value**_**b**_	**High**_**a**_	**%**	**p**-**value**_**b**_
total	13 / 25	52.0		15 / 24	62.5		11 / 26	42.3	
CCS I	2 / 9	22.2		4 / 10	40.0		1 / 9	11.1	
CCS II	7 / 10	70.0		6 / 8	75.0		7 / 10	70.0	
CCS III	4 / 6	66.7		5 / 6	83.3		3 / 6	50.0	
high grade_**c**_	11 / 16	68.8	**0**.**04**	11 / 14	78.6	0.09	10 / 16	62.5	**0**.**03**

a: number of tumor samples with high expression defined as showing a total sum score higher than 3.

b: p-value of the Fisher’s exact test for the comparison of the corresponding sample group with CCS I. Significant p-values are shown in bold.

c: high grade: grade II and grade III.

### Expression of the co-receptor endoglin

Endoglin / CD105 is a TGFβ co-receptor with the ability to modulate TGFβ signaling through Smad1/5/8 or Smad2/3 in various cell types including chondrocytes [7,8,15]. In order to establish whether endoglin could influence TGFβ signaling in chondrosarcoma, we have assessed its expression in chondrosarcoma by immunohistochemical analysis. Endoglin is an established marker of tumor vasculature [16]. Endoglin was detected in the cytoplasm and on the membrane of tumor and vascular cells. Only expression in tumor cells and not in the vasculature was scored in this study (Figure 2C). Only one grade I chondrosarcoma showed a sum score for endoglin higher than 3 and high endoglin expression was significantly more frequent in high-grade tumors (Table 1). From the 10 chondrosarcoma samples with high endoglin expression, 9 showed endoglin expression in more than 50% of tumor cells. There was a trend close to significance for a shorter metastasis-free survival in patients with high endoglin expression in more than 50% of the tumor cells (p=0.052) (Figure 2E). This correlation was not independent from the histopathological grade of the tumors. Notably, among the samples with low endoglin expression only 33% showed highly phosphorylated Smad1/5/8 while from the samples with high endoglin expression more than 80% also showed highly phosphorylated Smad1/5/8 (Figure 2F). High endoglin expression correlated with highly phosphorylated Smad1/5/8 (p=0.036, Pearson’s chi-square test) but not with highly phosphorylated Smad2.

### Activity of Smad1 and Smad2 in chondrosarcoma cell lines

**Figure 3:**
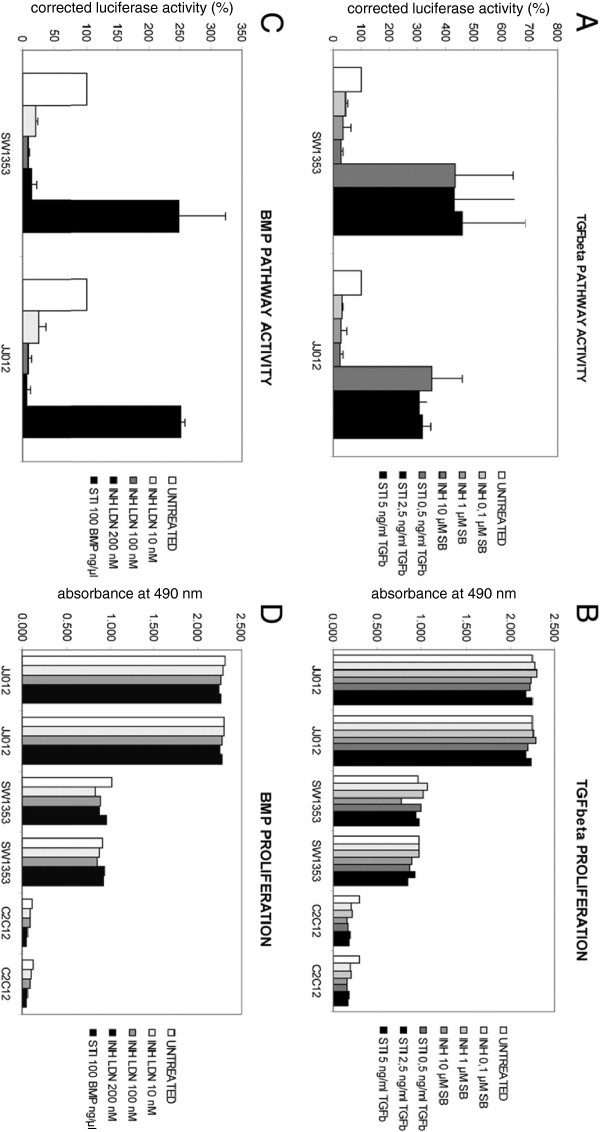
**TGFβ and BMP pathway activity and its effect on proliferation.A**: TGFβ pathway activity assayed using CAGA-luc reporter assay. The Y-axis is in percentage of luciferase activity with untreated cells set to 100%. Cells were treated with three different concentrations of the TGFβ inhibitor SB-431542 or with TGFβ1, to stimulate the pathway **B**: Proliferation of chondrosarcoma cells is not affected by treatment with TGFβ inhibitor or stimulator in two chondrosarcoma cell lines, nor in C2C12 cells. **C**: BMP pathway assayed using BRE-luc reporter assay, shown as in A. Inhibition of BMP with three different concentrations of LDN-193189 or stimulation with BMP4. **D**: Proliferation of chondrosarcoma cells is not affected by manipulation of BMP pathway activity.

Functional activity of the TGFβ- and BMP pathways was tested in the chondrosarcoma cell lines SW1353 and JJ012 using luciferase reporter assays with two reporter plasmids carrying pSmad2 (CAGA-luc) and pSmad1 (BRE-luc) responsive promoter elements (Figure 3). Pathway activity was shown by activation of the luciferase reporter genes, as shown by bioluminescence. Bioluminescence intensity could be inhibited by specific inhibitors, SB-431542 for TGFβ (Figure 3A) or LDN-193189 for BMP (Figure 3C). Stimulation of the pathways could also be achieved by TGFβ1 (Figure 3A) or BMP4 (Figure 3C). There was more variation in SW1353 than JJ012 in stimulation of both pathways when comparing three separate assays. Despite responsiveness of chondrosarcoma cells to specific manipulation of TGFβ and BMP activity there was no effect on proliferation of the cells upon inhibition or stimulation of the pathways (Figure 3B, D).

## Discussion

We have shown for the first time that the BMP signaling pathway is active in conventional central chondrosarcoma and that the activity correlates to the histopathological grade of the tumors as there were significantly more high-grade than low-grade chondrosarcomas with highly nuclear phosphorylated Smad1/5/8. Nuclear phosphorylated Smad2 was also detected but did not correlate to grade. Activity of both signaling pathways was furthermore confirmed through functional assays in 2 chondrosarcoma cell lines. Both pathways were found to be inducible upon stimulation with TGFβ1 or BMP4. Interestingly, changes in pathway activity did not affect cell proliferation.

Smad1/5/8 activation can on one hand be driven by BMPs through the ALK1/2/3/6 receptors. Our gene expression analysis of BMPs suggests that transcriptional regulation of BMPs is not relevant for the progression of chondrosarcoma. Higher expression of the type I receptor ALK2 in high-grade chondrosarcoma could however contribute to enhanced BMP signaling and phosphorylated Smad1/5/8 in these tumors compared to grade I. On the other hand, Smad1/5/8 can also be activated by TGFβ driven ALK1 activation as it has been shown in endothelial cells, neurons, hepatic stellate cells and chondrocytes [7]. In that case, elevated TGFβ3 expression in grade III chondrosarcoma compared to grade I could contribute to Smad1/5/8 activation in these tumors. Our gene expression profiles suggest that the BMP and TGFβ signaling pathways are regulated very differently between normal cartilage and chondrosarcoma. As the crosstalk between TGFβ and BMP signaling pathways is known to be highly context-dependent [17], it should be elucidated whether mechanisms described in chondrocytes could also be relevant in chondrosarcoma cells. This could be performed in the chondrosarcoma cell lines, for which we have shown activity of both signaling pathways.

In endothelial cells, it has been described that TGFβ/ALK5/Smad2/3 signaling antagonizes TGFβ/ALK1/Smad1, and that the balance of TGFβ/ALK1 versus TGFβ/ALK5 represents a determinant of the pro- and anti-angiogenic effects of TGFβ [7]. It has also been proposed that the ratio of ALK1/ALK5 expression is a determinant of TGFβ signaling in chondrocytes and that high ratios result in a stronger activation of Smad1/5/8 [18]. ALK5 was significantly lower expressed in chondrosarcoma in comparison to cartilage while expression levels of ALK1 were equal. The ALK1/ALK5 ratio in chondrosarcoma could thus favor Smad1 activation in comparison to normal cartilage. Smad1/5/8 signaling is strongly associated with chondrocyte terminal differentiation and hypertrophy [18]. Transgenic mouse models have shown that a deletion of Smad1 and Smad 5 results in chondrodysplasia and inhibition of the differentiation of proliferating chondrocytes [19,20]. However, in chondrosarcoma no hypertrophic differentiation occurs and we have observed that phosphorylated Smad1/5/8 was elevated in high-grade tumors with a less differentiated phenotype. Other mechanisms such as elevated PTHrP signaling in chondrosarcoma may be blocking hypertrophy in these tumors [21].

The TGFβ co-receptor endoglin has been described as a central modulator of these signaling pathways in endothelial cells and chondrocytes [7,8]. In human articular chondrocytes, endoglin interacts with ALK1 [22] and was shown to enhance TGFβ1-induced Smad1/5 phosphorylation and to inhibit TGFβ1-induced Smad2 phosphorylation [8]. In central chondrosarcoma, we found significantly higher expression of endoglin in high-grade tumors and a correlation of endoglin expression to Smad1/5/8 activity. This correlation suggests that endoglin expression in high-grade chondrosarcoma could represent a determinant of elevated Smad1/5/8 activation in these tumors. This could involve TGFβ as well as BMP signaling, as in Ewing sarcoma and melanoma cell lines endoglin was shown to lead also to higher BMP induced Smad1 phosphorylation [23]. On the other hand, endoglin is not exclusively modulating the Smad1/5/8 activation. In bone marrow stromal cells, endoglin appears to be a positive regulator of both ALK1/Smad1/5/8 and ALK5/Smad2 pathways [24]. The dissection of signaling pathways in chondrosarcoma cells would be necessary to determine whether the correlation of endoglin expression to Smad1/5/8 phosphorylation in these cells truly reflects an enhanced activation of this signaling axis in high grade chondrosarcoma.

Endoglin / CD105 is one of the classical markers expressed by mesenchymal stem cells and used for the definition of these cells [25]. Endoglin expression is up-regulated during the dedifferentiation of chondrocytes [26] and conversely down-regulated during the chondrogenic differentiation of mesenchymal stem cells [27]. In bone marrow stromal cell lines, endoglin was shown to stimulate proliferation [24]. In this context, thus, endoglin and Smad1 signaling correlate to undifferentiated states of proliferating chondrogenic precursors, which is in line with higher expression levels in high-grade chondrosarcoma. Our reporter assay indicates that the Smad1 and Smad2 signaling pathways may not be relevant for proliferation of chondrosarcoma cells. Thus, while endoglin / Smad1 signaling seem important for loss of differentiation, it is not crucial for proliferation.

Endoglin has furthermore been described to have a pivotal function in vascular development and disease [28]. Endoglin expression is stimulated by hypoxia through the transcription factor HIF1α [29]. It is a marker of activated endothelial cells and its expression has been established as a specific marker for tumor endothelium in several tumor types [16]. Its expression was however not found exclusively in tumor endothelium but also in tumor cells in melanoma, ovary and prostate tumors [28] and now in chondrosarcoma. We have previously described a constitutive activation of HIF1α in high-grade chondrosarcoma as well as elevated expression of HIF1α target genes in these tumors [30]. The expression pattern of endoglin, as a further HIF1α target gene, is in line with these results. Therefore, the hypothesis can be made that endoglin could represent an important mediator of tumor angiogenesis in high-grade chondrosarcoma. It is known that high grade chondrosarcomas demonstrate increased microvessel density [30,31] and this phenomenon is also clinically used in dynamic MRI and to diagnose chondrosarcoma. A correlation between microvessel density and endoglin is therefore likely, but would not prove a causal relation between these two phenomena. An association between angiogenesis and endoglin expression could only be approached in vitro in chondrosarcoma cells and animal models.

Since central chondrosarcoma is a rare tumor type and the isolation of good quality RNA is difficult due to low cellularity and extracellular matrix [32], one limitation of this study is the restricted number of samples which allowed reaching only levels of significance close to the threshold. The analysis of larger patient groups would be necessary to establish the robustness of the correlations found in this study and would especially be interesting to assess whether high endoglin expression significantly correlates to a high tumor vascularization and to a low metastasis-free survival.

## Conclusions

We have shown that the BMP and TGFβ signaling pathways are active in conventional central chondrosarcoma and that phosphorylated Smad1/5/8 and endoglin expression were significantly higher in high-grade compared to low-grade chondrosarcoma and correlated to each other. This correlation suggests that, as described in other cell types, endoglin could enhance Smad1/5/8 signaling in high-grade chondrosarcoma cells. Endoglin expression coupled to Smad1/5/8 activation could thus represent a functionally important signaling axis for the progression of chondrosarcoma and possibly a regulator providing a link between the undifferentiated phenotype of tumor cells in high-grade chondrosarcoma and the angiogenic status of these tumors. From our study it appears that both ALK1 and ALK2 could be type I receptors implicated in this signaling axis. Pharmacological targeting of ALK1 in a mouse model for endocrine pancreatic tumorigenesis and of ALK2 in ovarian cancer has recently been proven to be able to reduce tumor growth and angiogenesis [33,34]. Our results indicate that targeting ALK1 or ALK2 in high-grade central chondrosarcoma could represent a strategy to induce differentiation and repress angiogenesis in these tumors.

## Methods

### Tissue samples

From a collection of 30 conventional central chondrosarcoma cases, 26 fresh frozen tumor samples from the archives of the Department of Pathology of the Leiden University Medical Center and from the tumor bank of the Orthopaedic University Hospital Heidelberg, including 10 grade I, 10 grade II and 6 grade III tumors, were available for gene expression analysis. For immunohistochemical analysis, from the same collection of central tumors, formalin-fixed, paraffin-embedded material from 27 cases including 10 grade I, 11 grade II and 6 grade III tumors was retrieved from the files of the Leiden University Medical Center. In 23 of the cases, both gene expression and immunohistochemical analysis were performed. Histological grading was performed for all cases according to Evans by the same pathologist to avoid interobserver variability [35]. Except for one case of Ollier disease, all chondrosarcomas analyzed were solitary. Fresh frozen normal articular cartilage samples (n=6) obtained from patients undergoing amputation were used as normal controls for gene expression analysis. Specimens from Leiden were handled according to the ethical guidelines described in "Code for Proper Secondary Use of Human Tissue in The Netherlands" of the Dutch Federation of Medical Scientific Societies. For the cases from Heidelberg, the study was approved by the local ethics committee (medical faculty of Heidelberg) and informed consent was obtained from all individuals included in the study.

### RNA isolation and quantitative real-time polymerase chain reaction

All tissue samples were processed centrally in one lab following the same protocol. Haematoxylin and eosin-stained frozen sections were used to ensure the presence of at least 70% of tumor cells in the material used for RNA isolation. Shock-frozen tumor and cartilage tissue was pulverized mechanically and consecutively dissolved in lysis/binding buffer for direct poly(A)^+^-mRNA isolation using oligo-d(T)-coupled beads (Dynabeads; Invitrogen). mRNA was subjected to first strand cDNA synthesis using reverse transcriptase (Sensiscript, Qiagen, Hilden, Germany) and oligo-d(T) primers. Expression levels of individual genes were analyzed by quantitative RT-PCR (Lightcycler, Roche). Aliquots of first-stranded cDNA were amplified using gene-specific primer sets (Table 2) obtained from Eurofins (Ebersberg, Germany) and real-time fluorimetric intensity of SYBR green I was monitored. The candidate normalization genes described for gene expression analysis of chondrosarcoma [21] SRPR, CPSF6, CAPNS1 and HNRPH1 were used as reference. For each gene, the number of cDNA copies was correlated with the apparent threshold cycle (Ct). Building the difference between Ct of the gene of interest and the mean Ct of the reference genes for each sample gave ∆Ct values that were expressed as a percentage of reference genes. Melting curves and agarose gel electrophoresis of the PCR products were used for quality control.

**Table 2:** **Primer sets used for quantitative RT**-**PCR analysis**

**Gene symbol**	**alias**	**forward primer(5'to 3')**	**reverse primer(5'to 3')**
BMP2		ACGAGGTCCTGAGCGAGTTC	GAAGCTCTGAGGTGATAA
BMP4		CACCTCATCACACGACTAC	GGCATGGTTGGTTGAGTTG
BMP6		GAGTCGTAATCGCTCTACCC	GTGAACCAAGGTCTGCACAA
BMP7		CCAGAACCGCTCCAAGAC	GTTGGTGGCGTTCATGTAG
TGFB1		CAGAAATACAGCAACAATTCC	CTGAAGCAATAGTTGGTGTC
TGFB2		CCAAAGGGTACAATGCCAAC	CAGATGCTTCTGGATTTATGGTATT
TGFB3		TAAGCGGAATGAGCAGAGG	CCACGCCTTTGAATTTGATTT
ACVRL1	ALK1	CTGGACATCGGCAACAAC	ACACCACCTTCTTCATGTC
ACVR1	ALK2	TGGCTTCCACGTCTACCA	GCGAACACTACAGAGAGAA
BMPR1A	ALK3	TATGGAGAAGTATGGATGGG	GAACCTGTACCTTTAATGTCT
TGFBR1	ALK5	ATTACCAACTGCCTTATTATGA	CATTACTCTCAAGGCTTCAC
BMPR2		TGGGATAGGTGAGAGTAGA	GAGGGAGGAGTGGTAGTT
TGFBR2		ATGGAGGCCCAGAAAGATG	GACTGCACCGTTGTTGTCAG

### Immunohistochemistry

Immunohistochemistry was performed as described previously [36]. Details of primary antibodies are described in Table 3. As negative controls, slides were incubated with PBS/BSA 1% instead of primary specific antibodies. An IHC protocol optimized for cartilaginous tissue was applied to avoid detaching of sections. Antigen retrieval was performed using citrate buffer, pH6.0 at 98°C for 10 minutes in a microwave followed by cooling down for 2 h. The antibodies were incubated over night at room temperature. They were visualized using the DAB+ substrate-chromogen system (Dako, Heverlee, Belgium).

**Table 3:** Antibodies used for immunohistochemical analysis

**Antigen**	**Manufacturer**	**Mono**-/**polyclonal**	**Staining**	**Positive control**	**Blocking**	**Concentration**
phospho-Smad1 (Ser463/465)	Cell signaling	polyclonal rabbit IgG	nucleus	colon	no	1:200
/Smad5 (Ser463/465)						
/Smad8 (Ser426/428)						
phospho-Smad2 (Ser465/467)	Cell signaling	monoclonal rabbit IgG1	nucleus	kidney	10% normal goat serum, 30’	1:50
endoglin / CD105	R&D systems	polyclonal goat IgG	cytoplasmic	tonsil	no	1:800

### Evaluation and scoring

Semi quantitative scoring of immunohistochemical staining for phosphorylated Smad1/5/8 (pSmad1/5/8), phosphorylated Smad2 (pSmad2) and endoglin was performed as described previously [36]. Slides were evaluated blinded towards clinicopathological data. In short, staining intensities (0 = negative, 1 = weak, 2 = moderate, and 3 = strong intensity) and the percentage of positive cells (0 = 0%, 1 = 1–24%, 2 = 25–49%, 3 = 50–74%, and 4 = 75–100% positive) were assessed. For statistical analysis slides were scored as “high expression” when the sum score of the staining intensity and the percentage of positive cells were greater than 3.

### Cell line typing

Early and late passages of the cell lines SW1353 [37] and JJ012 [38] were tested for their STR loci using the Powerplex CellIDTM system (Promega) in order to obtain a genetic profile. For SW1353, the genetic profiles according to these loci were identical to the profile submitted to the DSMZ database (http://www.dsmz.de). For JJ012 no genetic profile is submitted to the DSMZ database. Early and late passage had identical profiles and did not match with any other cell line in the DSZM database.

### Plasmids

The BMP-responsive element (BRE)-luciferase construct that drives a luciferase gene was obtained from Prof. ten Dijke [39]. The TGFβ pathway responsive plasmid containing (CAGA)12-luciferase reporter, which is exclusively activated by TGF-β-induced complex, has been described previously [40]. pRL-CAGGS expresses Renilla luciferase under a constitutive CAGGS promoter and was obtained from Promega.

### Manipulation of TGFÎ²- and BMP pathways

TGFβ activity is inhibited by SB-431542 (Tocris Bioscience) at different concentrations (0.1, 1 and 10 μM) and stimulated by TGFβ1 (Sigma) (0.5, 2.5 and 5 ng/ml). BMP activity is manipulated by LDN-193189 (Stemgent Inc.) (10, 100 and 200 nm) and BMP4 (R&D systems). Mouse osteoblastic cells C2C12 were used as positive control for TGFβ and BMP activity. Untreated and manipulated C2C12 cells showed luciferase reporter activity in the same range as chondrosarcoma cells.

### Proliferation assay

The number of viable cells was determined by using a Cell Titer-96 Aqueous One Solution Cell Proliferation Assay (MTS) from Promega, Madison, USA. Cells were seeded at a density of 2000 cells per well in 96-well flat-bottom plates. The next day, medium was replaced by fresh medium containing drug as indicated or DMSO, each condition in triplicate. The MTS assay was performed according to the manufacturer’s instructions and absorbance was measured at 490 nm using a Victor3 Multilabel Counter 1420–042 (Perkin Elmer, MA, USA).

### Transient transfection and luciferase assay

Cells were seeded at a density of 5000 cells per well in 96-well flat-bottom plates. Next day, 100μl transfection complex was prepared with 1.95 μg of each plasmid driving luciferase expression from the corresponding BMP or TGFβ responsive promoters and 0.05 μg of pRL-CAGGS, an internal control for transfection efficiency driving renilla expression from a constitutive promoter. 5μl of the mix was added per well using Fugene HD transfection reagent (Roche, Mannheim, Germany) according to the manufacturer’s protocol. After 24 hours the medium was replaced by medium supplemented with 300ng/ml BMP4 or 10, 100, 200nM LDN-193189. After 24 h incubation, cells were harvested and luciferase activity was measured with a Victor 3 Multilabel Counter 1420–042 using the Dual-luciferase Reporter Kit (Promega). The ratio of firefly to renilla fluorescence was calculated to normalize reporter activity to the transfection efficiency. Three independent transfections were performed, each in triplicate.

### Statistical analysis

Data analysis was performed with SPSS for Windows (SPSS, Chicago, USA). Median values of gene expression levels as assessed by quantitative RT-PCR were calculated. The Mann–Whitney test was chosen to evaluate significant differences in gene expression levels between sample groups. For the comparison of gene expression levels between chondrosarcoma of different grades and between cartilage samples and chondrosarcoma in Figure 1, the bonferroni correction was used and p<0.0125 was considered significant. For the analysis of immunohistochemical data, the Pearson chi-square test/Fisher’s exact test, two-sided was used for comparison between low- and high-grade chondrosarcoma. Since the number of samples of grade III chondrosarcoma (n=6) alone was considered too low for this test the clinically more relevant comparison between low-grade (grade I) and high-grade (grade II + III) chondrosarcoma was considered. Total survival and metastasis-free survival curves based on Kaplan–Meier estimates were compared using log rank test. For all tests a p value <0.05 was considered significant.

## Competing interests

The authors declare that they have no competing interests.

## Authors' contributions

SB participated in the design of the study, carried out the gene expression study, analyzed the data and drafted the manuscript. JVMGB conceived the study, participated in its design and coordination, and in the analysis of the data, helped to draft the manuscript. BL participated in the design of the study. BA and MR carried out the immunohistochemistry and the cell line assays. AMCJ participated in the design of the study and analyzed the cell line assays. WR conceived the study, and participated in its design and coordination, helped to draft the manuscript. All authors read and approved the final manuscript.

## Funding

The Research Centre for Experimental Orthopaedics and the Department of Pathology, Leiden University Medical Centre are partners of the EuroBoNeT consortium, a European Commission FP-6 granted Network of Excellence for studying the pathology and genetics of bone tumours.

## Pre-publication history

The pre-publication history for this paper can be accessed here:

http://www.biomedcentral.com/1471-2407/12/488/prepub

## References

[B1] Bertoni F Bacchini P Hogendoorn PCW Fletcher CDM, Unni KK, Mertens F Chondrosarcoma World health organisation classification of tumours. Pathology and genetics of tumours of soft tissue and bone 2002 Lyon: IARC Press 247 251

[B2] Bovee JVMG Hogendoorn PCW Wunder JS Alman BA Cartilage tumours and bone development: molecular pathology and possible therapeutic targets Nat Rev Cancer 2010 10 481 488 10.1038/nrc2869 20535132

[B3] Aigner T Towards a new understanding and classification of chondrogenic neoplasias of the skeleton–biochemistry and cell biology of chondrosarcoma and its variants Virchows Arch 2002 441 219 230 10.1007/s00428-002-0641-x 12242518

[B4] Boeuf S Kunz P Hennig T Lehner B Hogendoorn PCW Bovee JVMG Richter W A chondrogenic gene expression signature in mesenchymal stem cells is a classifier of conventional central chondrosarcoma J Pathol 2008 216 158 166 10.1002/path.2389 18702172

[B5] Kronenberg HM Developmental regulation of the growth plate Nature 2003 423 332 336 10.1038/nature01657 12748651

[B6] Miyazono K Kamiya Y Morikawa M Bone morphogenetic protein receptors and signal transduction J Biochem 2010 147 35 51 10.1093/jb/mvp148 19762341

[B7] Pardali E Goumans MJ ten Dijke P Signaling by members of the TGF-beta family in vascular morphogenesis and disease Trends Cell Biol 2010 20 556 567 10.1016/j.tcb.2010.06.006 20656490

[B8] Finnson KW Parker WL Chi Y Hoemann CD Goldring MB Antoniou J Philip A Endoglin differentially regulates TGF-beta-induced Smad2/3 and Smad1/5 signalling and its expression correlates with extracellular matrix production and cellular differentiation state in human chondrocytes Osteoarthr Cartil 2010 18 1518 1527 10.1016/j.joca.2010.09.002 20833252

[B9] Pogue R Lyons K BMP signaling in the cartilage growth plate Curr Top Dev Biol 2006 76 1 48 17118262 10.1016/S0070-2153(06)76001-X

[B10] Schrage YM Hameetman L Szuhai K Cleton-Jansen AM Taminiau AH Hogendoorn PCW Bovee JVMG Aberrant heparan sulfate proteoglycan localization, despite normal exostosin, in central chondrosarcoma Am J Pathol 2009 174 979 988 10.2353/ajpath.2009.080623 19179614 PMC2665757

[B11] Masi L Malentacchi C Campanacci D Franchi A Transforming growth factor-beta isoform and receptor expression in chondrosarcoma of bone Virchows Arch 2004 440 491 497 12021923 10.1007/s00428-001-0544-2

[B12] Yoshikawa H Nakase T Myoui A Ueda T Bone morphogenetic proteins in bone tumors J Orthop Sci 2004 9 334 340 10.1007/s00776-004-0764-9 15168194

[B13] Guo W Gorlick R Ladanyi M Meyers PA Huvos AG Bertino JR Healey JH Expression of bone morphogenetic proteins and receptors in sarcomas Clin Orthop Relat Res 1999 365 175 183 10627702 10.1097/00003086-199908000-00023

[B14] Hou CH Hsiao YC Fong YC Tang CH Bone morphogenetic protein-2 enhances the motility of chondrosarcoma cells via activation of matrix metalloproteinase-13 Bone 2009 44 233 242 10.1016/j.bone.2008.09.021 19038372

[B15] Velasco S Alvarez-Munoz P Pericacho M ten Dijke PT Bernabeu C Lopez-Novoa JM Rodriguez-Barbero A L- and S-endoglin differentially modulate TGFbeta1 signaling mediated by ALK1 and ALK5 in L6E9 myoblasts J Cell Sci 2008 121 913 919 10.1242/jcs.023283 18303046

[B16] Dallas NA Samuel S Xia L Fan F Gray MJ Lim SJ Ellis LM Endoglin (CD105): a marker of tumor vasculature and potential target for therapy Clin Cancer Res 2008 14 1931 1937 10.1158/1078-0432.CCR-07-4478 18381930

[B17] Guo X Wang XF Signaling cross-talk between TGF-beta/BMP and other pathways Cell Res 2009 19 71 88 10.1038/cr.2008.302 19002158 PMC3606489

[B18] van der Kraan PM Blaney Davidson EN van den Berg WB A role for age-related changes in TGFbeta signaling in aberrant chondrocyte differentiation and osteoarthritis Arthritis Res Ther 2010 12 201 10.1186/ar2896 20156325 PMC2875624

[B19] Keller B Yang T Chen Y Munivez E Bertin T Zabel B Lee B Interaction of TGFβ and BMP signaling pathways during chondrogenesis PLoS One 2011 6 e16421 10.1371/journal.pone.0016421 21297990 PMC3030581

[B20] Retting KN Song B Yoon BS Lyons KM BMP canonical Smad signaling through Smad1 and Smad5 is required for endochondral bone formation Development 2009 136 1093 1104 10.1242/dev.029926 19224984 PMC2668702

[B21] Rozeman LB Hameetman L van Wezel T Taminiau AHM Cleton-Jansen AM Hogendoorn PCW Bovee JVMG cDNA expression profiling of chondrosarcomas: Ollier disease resembles solitary tumours and alteration in genes coding for components of energy metabolism occurs with increasing grade J Pathol 2005 207 61 71 10.1002/path.1813 16007578

[B22] Finnson KW Parker WL ten Dijke P Thorikay M Philip A ALK1 opposes ALK5/Smad3 signaling and expression of extracellular matrix components in human chondrocytes J Bone Miner Res 2008 23 896 906 10.1359/jbmr.080209 18333754

[B23] Pardali E van der Schaft DW Wiercinska E Gorter A Hogendoorn PC Griffioen AW ten Dijke P Critical role of endoglin in tumor cell plasticity of Ewing sarcoma and melanoma Oncogene 2011 30 334 345 10.1038/onc.2010.418 20856203

[B24] O'Connor JC Farach-Carson MC Schneider CJ Carson DD Coculture with prostate cancer cells alters endoglin expression and attenuates transforming growth factor-beta signaling in reactive bone marrow stromal cells Mol Cancer Res 2007 5 585 603 10.1158/1541-7786.MCR-06-0408 17579118

[B25] Dominici M Le Blanc K Mueller I Slaper-Cortenbach I Marini F Krause D Deans R Keating A Prockop D Horwitz E Minimal criteria for defining multipotent mesenchymal stromal cells The International Society for Cellular Therapy position statement. Cytotherapy 2006 8 315 317 10.1080/14653240600855905 16923606

[B26] Diaz-Romero J Gaillard JP Grogan SP Nesic D Trub T Mainil-Varlet P Immunophenotypic analysis of human articular chondrocytes: changes in surface markers associated with cell expansion in monolayer culture J Cell Physiol 2005 202 731 742 10.1002/jcp.20164 15389573

[B27] Lee HJ Choi BH Min BH Park SR Changes in surface markers of human mesenchymal stem cells during the chondrogenic differentiation and dedifferentiation processes in vitro Arthritis Rheum 2009 60 2325 2332 10.1002/art.24786 19644865

[B28] ten Dijke P Goumans MJ Pardali E Endoglin in angiogenesis and vascular diseases Angiogenesis 2008 11 79 89 10.1007/s10456-008-9101-9 18283546

[B29] Sanchez-Elsner T Botella LM Velasco B Langa C Bernabeu C Endoglin expression is regulated by transcriptional cooperation between the hypoxia and transforming growth factor-beta pathways J Biol Chem 2002 277 43799 43808 10.1074/jbc.M207160200 12228247

[B30] Boeuf S Bovee JVMG Lehner B Hogendoorn PCW Richter W Correlation of hypoxic signalling to histological grade and outcome in cartilage tumours Histopathology 2010 56 641 651 10.1111/j.1365-2559.2010.03528.x 20459575

[B31] Ayala G Liu C Nicosia R Horowitz S Lackman R Microvasculature and VEGF expression in cartilaginous tumors Hum Pathol 2000 31 341 346 10.1016/S0046-8177(00)80248-8 10746677

[B32] Baelde HJ Cleton-Jansen AM van Beerendonk H Namba M Bovée JVMG Hogendoorn PCW High quality RNA isolation from tumours with low cellularity and high extracellular matrix component for cDNA microarrays: application to chondrosarcoma J Clin Pathol 2001 54 778 782 10.1136/jcp.54.10.778 11577126 PMC1731295

[B33] Cunha SI Pardali E Thorikay M Anderberg C Hawinkels L Goumans MJ Seehra J Heldin CH ten Dijke P Pietras K Genetic and pharmacological targeting of activin receptor-like kinase 1 impairs tumor growth and angiogenesis J Exp Med 2010 207 85 100 10.1084/jem.20091309 20065063 PMC2812548

[B34] Herrera B van Dinther M ten Dijke P Inman GJ Autocrine bone morphogenetic protein-9 signals through activin receptor-like kinase-2/Smad1/Smad4 to promote ovarian cancer cell proliferation Cancer Res 2009 69 9254 9262 10.1158/0008-5472.CAN-09-2912 19996292 PMC2892305

[B35] Evans HL Ayala AG Romsdahl MM Prognostic factors in chondrosarcoma of bone: a clinicopathologic analysis with emphasis on histologic grading Cancer 1977 40 818 831 10.1002/1097-0142(197708)40:2<818::AID-CNCR2820400234>3.0.CO;2-B 890662

[B36] Rozeman LB Hameetman L Cleton-Jansen AM Taminiau AHM Hogendoorn PCW Bovee JVMG Absence of IHH and retention of PTHrP signalling in enchondromas and central chondrosarcomas J Pathol 2005 205 476 482 10.1002/path.1723 15685701

[B37] Ottaviano L Schaefer KL Gajewski M Huckenbeck W Baldus S Rogel U Mackintosh C de Alava E Myklebost O Kresse SH Meza-Zepeda LA Serra M Cleton-Jansen AM Hogendoorn PC Buerger H Aigner T Gabbert HE Poremba C Molecular characterization of commonly used cell lines for bone tumor research: a trans-European EuroBoNet effort Genes Chromosomes Cancer 2010 49 40 51 10.1002/gcc.20717 19787792

[B38] Ghert MA Jung ST Qi W Harrelson JM Erickson HP Block JA Scully SP The clinical significance of tenascin-C splice variant expression in chondrosarcoma Oncology 2001 61 306 314 10.1159/000055338 11721178

[B39] Korchynskyi O ten Dijke P Identification and functional characterization of distinct critically important bone morphogenetic protein-specific response elements in the Id1 promoter J Biol Chem 2002 277 4883 4891 10.1074/jbc.M111023200 11729207

[B40] Dennler S Itoh S Vivien D ten Dijke P Huet S Gauthier JM Direct binding of Smad3 and Smad4 to critical TGF beta-inducible elements in the promoter of human plasminogen activator inhibitor-type 1 gene EMBO J 1998 17 3091 3100 10.1093/emboj/17.11.3091 9606191 PMC1170648

